# Management of Guttate Psoriasis: A Systematic Review

**DOI:** 10.1177/12034754241266187

**Published:** 2024-07-30

**Authors:** Ted Zhou, John Koussiouris, Lauren Kim, Ronald Vender

**Affiliations:** 1 Department of Medicine, McMaster University, Hamilton, ON, Canada; 2 Division of Dermatology, Department of Medicine, McMaster University, Hamilton, ON, Canada

**Keywords:** psoriasis, treatment, clinical dermatology, systematic review

## Abstract

Guttate psoriasis (GP) is a variant of psoriasis characterized by scattered “drop-like” papules and plaques, accounting for up to a quarter of psoriasis cases. Although GP can clear within 3 to 4 months, up to 39% of cases may progress to chronic plaque psoriasis. Currently, there is a paucity of literature investigating the efficacy of different treatment modalities. This systematic review aims to synthesize all available data on GP treatment efficacy. A literature search was conducted using Medline, Embase, Web of Science, and CINAHL with no date limits. A total of 75 studies satisfied eligibility criteria and were analyzed. Most studies were case reports, series, or retrospective studies. Only 5 randomized controlled trials (RCTs) were identified. For topical treatments, corticosteroids and calcipotriol creams had the most evidence for efficacy. Four categories of systemic therapies were identified: traditional immunosuppressants, antibiotics, retinoids, and biologics. Evidence regarding antibiotic therapy suggests minimal connection between underlying infection resolution and GP lesion remission. Phototherapy had the most robust evidence, with narrowband ultraviolet B (UVB) being the most effective. Our findings are limited by high heterogeneity in study design and high risk of bias. Based on our review, we propose the following treatment algorithm. As first-line therapy, we recommend topical corticosteroids and calcipotriol cream, in combination with phototherapy. As supportive therapy, we recommend antibiotics if applicable. For second-line therapy, we recommend methotrexate or cyclosporine. For severe and refractory GP, biologics can be used as third-line treatment. RCTs are needed to provide higher quality evidence to create standardized treatment recommendations.

## Introduction

Guttate psoriasis (GP) accounts for as many as a quarter of psoriasis cases.^1,2^ It characteristically presents in children and young adults as an acute eruption of numerous small, erythematous, scaly papules, mainly on the trunk and extremities.^3^ The outbreak may present as a novel disorder in individuals without a prior psoriasis history or may complicate existing chronic plaque-type psoriasis.^4^ The pathophysiology of GP is likely influenced by both genetic and environmental factors that contribute to an abnormal immune response in the skin. The human leukocyte antigen HLA-Cw6 and preceding streptococcal infection have both been associated with the disease.^3,5^ Cross-reactivity between streptococcal M-proteins and structurally similar antigens on human keratinocytes is thought to trigger an immune response.^5^ Although GP generally spontaneously remits 12 to 16 weeks after onset, as many as 39% of guttate cases may progress to chronic plaque psoriasis.^3^

Management options for GP include ultraviolet phototherapy, topical corticosteroids, topical vitamin D analogues, and systemic antibiotic therapy.^4^ Tonsillectomy has been suggested to be useful for recurrent episodes, due to the association between streptococcal infection and GP.^4^ Recently, systemic biological treatments have begun to be used for psoriasis.^6^ Unlike other types of psoriasis, literature reviews evaluating the efficacy of different management strategies for GP are fairly limited. The most widely cited systematic review investigating randomized control trials for GP was performed in 2001.^4^ The review identified only one randomized trial.^7^ A more recent Cochrane review found no additional randomized control trials that assessed the effects of non-antistreptococcal interventions for GP.^6^

To better inform clinical practice and management of GP, we conducted a systematic review of all available data in literature reporting on treatment efficacy. The primary outcome was to synthesize and assess the efficacy of all interventions for the management of pediatric and adult GP. Our secondary outcome was to report on the progression of GP to chronic plaque psoriasis.

## Materials and Methods

This review was conducted in accordance with guidelines set by the Preferred Reporting Items for Systematic Reviews and Meta-Analysis (PRISMA).^8^ Our protocol has been registered on PROSPERO (CRD42023481219).

### Outcomes

The primary outcome was to assess the efficacy of treatments for GP. Magnitude of response was divided into 4 categories: no response (NR), partial response (PR), significant response (SR), and not specified (NS).

For studies that did not specify an outcome assessment tool, treatment response was classified based on manuscript descriptions. The outcome assessment method was “physician’s judgment.” SR was defined as: “almost complete clearance,” “complete clearance,” “significant response,” “excellent response,” or a “marked response” after treatment. PR was defined as: a “moderate response” or “partial response” to treatment. NR was defined as: “no response” or “minimal response” or “poor response” to treatment.

For studies that specified an outcome assessment tool, treatment response was classified based on participant score improvement from baseline. SR was defined as: a ≥75% improvement of the patient’s score from baseline. PR was defined as: a ≥25% to ≤75% improvement. NR was defined as: a <25% improvement.

The secondary outcome was to investigate the progression of GP to chronic plaque psoriasis. This was done by reporting on the percentage of GP patients who developed plaque psoriasis.

### Literature Search

Databases used were MEDLINE (via OVID), Embase (via OVID), Web of Science, and CINAHL (via EBSCOhost) on September 21, 2023, with no date limits. The keywords in our search strategy were “psoriasis” and “guttate.” Subject Headings for “psoriasis,” “streptococcal infections,” and “interleukin-17” were included when possible. See Supplemental Table S1 for the search strategy. The references of included studies were reviewed to identify additional studies.

### Study Identification and Selection

A total of 2089 studies were returned by our search strategy. Duplicate studies were highlighted automatically by EndNote, a citation managing software, and were manually de-duplicated. Title, abstract, and full-text screening were performed separately by 2 independent reviewers (T.Z. and J.K.). Conflicts were resolved through discussion. A third reviewer (R.V.) acted as a third opinion if screening discrepancies could not be resolved.

### Inclusion and Exclusion Criteria

Studies were included if: (a) study included treatment outcome data for GP.

Studies were excluded if: (a) treatment outcome data for GP participants could not be identified; (b) only an abstract or conference proceeding could be found; (c) article was not in English; (d) study was a literature review.

### Data Collection and Extraction

Data were extracted independently by 2 separate reviewers (L.K. and J.K.) in-duplicate. A piloted, standardized data collection form was used. With the aid of a third reviewer (T.Z.), data were synthesized through consensus and any discrepancies were resolved. Characteristics extracted from all studies can be found in Supplemental Table S2.

### Risk of Bias in Individual Studies

The evaluation of risk of bias was conducted using “ROBINS-I” for nonrandomized studies and “RoB 2” for randomized trials.^9,10^ The outlined tools were employed by 3 separate reviewers (T.Z., J.K., and L.K.), and a consensus was reached for each domain and for the overall risk of bias. Any disagreements were resolved by a fourth reviewer (R.V.).

### Data Synthesis

Descriptive and quantitative analyses were performed when reporting on study characteristics and treatment efficacy. Tabular methods were used to display study results.

### Confidence in Cumulative Evidence

Certainty assessments of studies were not conducted due to the heterogeneity of study outcomes, study design, and the lack of effect sizes found.

## Results

Seventy-five studies were included in this review. A PRISMA diagram outlining study identification and screening is represented in Supplemental Figure S1.

### Case Reports and Series (Physician’s Judgment)

A total of 48 case reports and series were identified. See Supplemental Table S1 for a breakdown of treatment efficacy.

A total of 18 case reports investigated topical treatments. The most reported therapies were corticosteroids (n = 11),^11-21^ corticosteroids with topical calcipotriol (n = 4),^22-25^ corticosteroids with coal tar (n = 1),^26^ salicylic acid (n = 1),^27^ calcipotriene cream (n = 1).^28^ Of the 18 individuals receiving topical therapies, 15 achieved SR (83.3%) and 3 achieved PR (16.7%).

A total of 6 case reports and 2 case series investigated systemic antibiotic treatments. The reported therapies were unspecified antibiotic (n = 1),^29^ erythromycin monotherapy (n = 5),^30-32^ penicillin monotherapy (n = 6),^33-35^ penicillin with erythromycin (n = 2),^30,33^ and doxycycline (n = 1).^36^ All 15 patients receiving antibiotics achieved SR (100%).

A total of 6 case reports investigated systemic treatments for GP. The treatments were methotrexate (n = 3), cyclosporine (n = 1), and unspecified corticosteroids (n = 2). For patients on methotrexate, 1 achieved SR (33.3%), 1 achieved PR (33.3%), and 1 was NS (33.3%).^37-39^ For the 1 patient on cyclosporine, SR was achieved.^40^ Of the 2 patients on systemic corticosteroids, 1 achieved SR (50%) and 1 was NS (50%).^41,42^

A total of 6 case studies investigated phototherapy with or without other agents. The studied combinations were phototherapy with oral penicillin (n = 1),^43^ with topical corticosteroids (n = 4),^44-47^ and with retinoid cream (n = 1).^48^ Light modalities were narrowband ultraviolet B (UVB, n = 4 studies), psoralen and ultraviolet A (PUVA, n = 1 study), and unspecified (n = 1 study). All 6 individuals achieved SR (100%).

A total of 3 case studies and 1 case series reported on biologics. The agents were ustekinumab (n = 7),^49,50^ guselkumab (n = 1),^51^ and brodalumab (n = 1).^52^ All individuals treated with ustekinumab and guselkumab achieved SR (100%). Treatment response was unspecified for brodalumab.

Tonsillectomy was reported in 1 case study and 2 case series.^53-55^ Approximately 88.9% (n = 8 participants) achieved SR post-surgery, and 11.1% (n = 1 participants) achieved PR.

There was one reported case of complete GP resolution after autologous stem-cell rescue for a pediatric patient with Ewing’s sarcoma.^56^ One case report outlined the use of IVIG in a pediatric patient, who achieved a treatment response of SR.^57^ Another case report detailed a GP patient receiving an antifungal agent, fluconazole, for an underlying *Pityrosporum ovale* infection.^58^ Treating the infection led to a SR of GP symptoms, resolving ≥75% of lesions.

### Case Reports and Series (Outcome Measurement Tools)

A total of 3 case series and 5 case reports employed outcome measurement tools. The tools employed were Psoriasis Area and Severity Index (PASI, n = 5 studies), body surface area (BSA, n = 3 studies), and Physician Global Assessment (PGA, n = 2 studies). Individuals receiving risankizumab (n = 5),^59,60^ secukinumab (n = 3),^61,62^ and ixekizumab (n = 4)^61,63^ reported SR (100%) to treatment. Of the 2 individuals on guselkumab, one achieved SR (50%)^64^ and the other had NR (50%).^65^ One study reported on a single participant treated with PUVA, achieving SR.^66^

See Supplemental Table S2 for a breakdown.

### Case-Control

A 5 year case-control study followed GP patients positive for antistreptolysin O titer treated with or without a single course of oral antibiotics.^67^ Follow-up after 8 weeks found no statistically significant difference in time to clearance of GP lesions between the 2 groups.

### Cohort Studies

A total of 10 cohort studies were identified. Two prospective cohort studies reported on individual-level data, investigating the use of narrowband UVB phototherapy for treatment of GP. Of the 79 adults included, 64 achieved SR (81%), 10 achieved PR (12.7%), and 5 were NS (6.3%).^68,69^ One of these studies, by Fernandez-Guarino et al, identified variables associated with poor response to phototherapy.^68^ These were: older age of onset, longer time until treatment, higher PASI at baseline, and number of sessions needed.

A 10 year cohort study of 41 GP patients treated with phototherapy found that both broadband UVB and topical PUVA therapy resulted in SR for all patients based on BSA score improvement after a mean of 15.6 and 12.8 sessions, respectively.^70^ One cohort study with 20 patients treated by narrowband UVB showed an average PASI improvement of 73.7%.^71^

One prospective cohort study of 31 psoriasis patients included 1 patient with GP.^72^ This patient had SR to topical methoxsalen with UVA phototherapy. Another study investigating PUVA had 2 GP patients achieve SR assessed by PASI.^73^

One prospective study reported on topical and systemic retinoic acid.^74^ Two GP patients achieved SR, while 5 individuals had NR.

Two prospective cohort studies investigated oral rifampicin. One study employed a 2 week course of oral rifampicin to treat GP patients with unconfirmed previous streptococcal infection.^75^ Another study investigated rifampicin efficacy in a cohort of patients with or without confirmed concurrent streptococcal infection.^76^ Of 132 participants, 79 achieved SR (59.8%), 27 had PR (20.4%), 14 had NR (10.6%), and 12 were NS (9.1%).

One retrospective study investigated efficacy of systemic treatments.^77^ In total, 19 participants received acitretin, 6 received methotrexate, 7 received cyclosporine, and 3 individuals were on unspecified biologics. On acitretin, 6 achieved SR (31.6%), 3 achieved PR (15.8%), and 10 were NS (52.6%). On methotrexate, 2 achieved SR (33.3%), 3 achieved PR (50%), and 1 was NS (16.7%). On cyclosporine, 6 achieved SR (85.7%) and 1 was NS (14.3%). Last, 1 individual on biologics achieved SR (50%) and 1 achieved PR (50%).

### Randomized Controlled Trials

Three randomized controlled trials (RCTs) investigated GP patients’ response to antibiotics. One study of 43 adult GP patients showed no statistically significant clinical improvement after a 14 day clinical course of oral erythromycin or benzathine phenoxymethylpenicillin, compared to the control group.^78^ Another study of 20 adult GP patients was composed of 2 groups both treated with betamethasone dipropionate 0.05% cream and UVB therapy.^79^ One group was also treated with penicillin whereas the other was not. After 8 weeks, there was no statistically significant difference in PASI score between the 2 groups. In addition, a study of 92 GP patients found no significant difference in therapeutic response after 60 days of oral rifampicin in GP patients with or without concomitant streptococcal infections.^80^ However, there was statistically significant clinical improvement in patients receiving rifampicin compared to placebo.

Two additional RCT studies have compared other GP management options including phototherapy and lipid emulsions. A study by Boztepe et al assigned 14 GP patients who had achieved clinically significant improvement to either 2 months of narrowband UVB maintenance or no maintenance.^81^ After 1 year, there was no statistically significant difference in PASI scores between the 2 groups. A separate study of 20 GP patients were treated with either intravenous n-3 fatty acid emulsion or intravenous n-6 fatty acid emulsion.^7^ After 10 days, the n-3 group improved clinically significantly more than the n-6 group based on the physician’s judgment.

All RCTs are summarized in Supplemental Table S3.

### Progression of GP

Galili et al conducted a retrospective study of 120 GP patients who received diverse therapeutic interventions.^82^ At the conclusion of the follow-up period (mean: 6.2 years), 49.1% of patients exhibited enduring GP lesions and 17.5% of patients converted to psoriasis vulgaris. Two additional retrospective studies were analyzed, showing that 25.3% to 38.9% of GP cases progress to a chronic plaque psoriasis type.^3,83^

### Risk of Bias

The results of the risk of bias assessment for included studies can be found in Supplemental Tables S4 and S5. All included observational studies generally demonstrated “moderate” or “serious” risk of bias. The majority of RCTs had “high” risk of bias.

## Discussion

Our review summarizes all available evidence on the efficacy of topical, systemic, and phototherapy treatment options for GP. Based on our findings, there currently exists high heterogeneity in study designs, and a paucity of high-quality retrospective and prospective studies. The majority of RCTs investigating GP treatments have potential “high” risk of bias. This lack of data impedes the ability for clinicians to create evidence-based recommendations and makes it difficult to make definitive conclusions on treatment effectiveness. Progression of GP to a chronic plaque psoriasis type was found to range from 17.5% to 38.9%.

The most reported topical treatments that demonstrated effective remission of GP lesions were corticosteroids and calcipotriol creams. These 2 therapies seem to be effective in treating GP flares, especially in clinical scenarios where lesions are not widespread. However, this finding is based on 18 case reports, and is limited by the remission rates of GP and the fact that no RCT or cohort study investigated topical treatments.^82^ Based on available evidence, topical therapies should be employed as first-line, given favourable efficacy and ease of use. However, this may be difficult or impractical if lesions are widespread.

For systemic treatments, our review identified 4 categories: traditional immunosuppressants, antibiotics, retinoids, and biological agents. Immunosuppressants were sparsely reported, with 4 case reports and 1 retrospective cohort study. Based on the paucity of evidence in literature, traditional immunosuppressants, namely methotrexate and cyclosporine, should be reserved for refractory GP, or for widespread lesions impractical to treat with a topical-only approach.

The efficacy of antibiotic therapy remains a topic of discussion. The most common antibiotics used for GP were penicillin or erythromycin or rifampicin, with or without concurrent topical treatments. In case reports and series, all patients achieved SR. Among 2 cohort studies, a 59.8% SR achievement rate was reported. Despite this promising evidence, other studies cast doubt on the true effectiveness of antibiotics. One case-control study and 2 RCTs that investigated antibiotic therapies in patients with suspected streptococcal infection found no statistically significant differences in clearance time or magnitude of response.^67,78,79^ It is well-established that underlying infections, such as streptococcal infections, are highly associated with GP eruptions.^84^ It is still unclear, based on clinical data, that the resolution of underlying infection also directly alleviates GP lesions. Our review underlines conflicting data on the efficacy of antibiotics in treating GP. Given that patients generally benefit from infection remission, clinicians should consider the use of antibiotics as supportive therapy, when possible, in GP patients with underlying infection. However, clinicians should consider that there is minimal evidence suggesting a connection between underlying infection resolution and improvement of GP lesions.

Although retinoids have been described to be an effective therapeutic option for psoriasis subtypes like pustular and plaque, there remains little evidence for GP. Only one cohort study reported a 31.6% SR achievement rate in GP patients treated with acitretin.^77^ Unlike other psoriasis types, there are not enough robust data to evaluate or recommend retinoids as an effective treatment for GP.

Biologics, primarily anti-IL-17, anti-IL-23, and anti-IL-12/23 inhibitors, were effective in treating GP. This reflects current biologic practice for plaque psoriasis. However, this is based on only case reports and series. A recent network analysis of biologics for psoriasis illuminated that many clinical trials specifically exclude GP from their studies, possibly due to its natural course.^85^ This makes it difficult to fairly evaluate their effectiveness for GP. Moreover, even with promising preliminary data regarding biologics, cost and insurance coverage remains a significant accessibility barrier.^86,87^ It remains impractical to use biologics for the average GP patient, and we recommend that clinicians defer the use of biologics for long-standing, refractory GP, or when other therapies are contraindicated.

Phototherapy is a promising treatment option highlighted by our review. All individuals receiving phototherapy in case reports and series achieved SR. Moreover, 5 cohort studies showed efficacy of both narrow-band UVB and PUVA phototherapies. There were no clinically significant side effect profiles noted by any study. Our review suggests that phototherapy, both narrow-band UVB and PUVA, are effective as a first-line therapy for GP, similar to the plaque psoriasis subtype.^88^ On the other hand, maintenance phototherapy to prevent relapse of GP lesions appears to be ineffective. Only one RCT investigated maintenance treatment with narrow-band UVB for resolved GP and found no statistically significant difference in relapse rates.

The transition of GP to a chronic plaque psoriasis subtype is of clinical interest, especially if early intervention can decrease the risk of development. Our review identified 3 cohort studies that investigated relapse of GP after resolution, and the percentage of patients that progressed to a plaque subtype. Progression of GP to a plaque psoriasis subtype ranged from 17.5% to 38.9%. Treatment of GP should consider the fact that GP is associated with lower quality of life in patients with psoriasis, causing psychological distress potentially due to its acute onset.^11,89^ It remains unclear if early GP remission, or effective treatment, may decrease the risk of progression to a chronic, plaque psoriasis.^82^ Additional research is required to assess whether certain treatments and time to resolution of GP lesions predict progression. This would help inform clinicians on how to approach management, especially in severe cases.

The risk of bias for both nonrandomized and randomized studies were found to be either “moderate” or “serious.” Specifically, for RCTs, 4 out of 5 studies had “high” overall risk of bias. Moreover, the included studies had high heterogeneity in study design and treatments investigated. Overall, the quality of evidence to base treatment recommendations remains uncertain. This finding is supported by similar studies investigating GP treatment efficacy.^90^ Clinicians should be aware of this deficit in psoriasis literature, and higher quality studies are needed to better inform on evidence-based management approaches for GP.

### Limitations

We did not perform any meta-analysis of treatment response, primarily due to the heterogeneity in study designs and treatments reported. Moreover, we only included English articles, which may have affected results.

## Conclusion

Our review synthesizes all available treatment data for GP in literature, highlighting a variety of treatment modalities with promising efficacy. Most studies investigating treatment efficacy were case reports, series, and retrospective cohort studies. Based on our findings, we propose the following treatment algorithm:

*First-line therapy*: Topical corticosteroids, calcipotriol cream, phototherapy (narrow-band UVB or PUVA).
*Supportive therapy*: Antibiotics if an underlying infection is suspected (ie, perianal streptococcal infection or streptococcal pharyngitis).
*Second-line therapy*: Methotrexate, cyclosporine.
*Third-line therapy*: Biologics (anti-IL-17, anti-IL-23, anti-IL-12/23).

Our review stresses the need for additional studies that investigate treatment modalities for GP, and risk factors for progression to a chronic plaque psoriasis subtype. Currently, there is a paucity of high-quality evidence to base recommendations, and clinicians should use their judgment and experiences to help inform their treatment approach.

## Supplemental Material

sj-docx-1-cms-10.1177_12034754241266187 – Supplemental material for Management of Guttate Psoriasis: A Systematic Review Supplemental material, sj-docx-1-cms-10.1177_12034754241266187 for Management of Guttate Psoriasis: A Systematic Review by Ted Zhou, John Koussiouris, Lauren Kim and Ronald Vender in Journal of Cutaneous Medicine and Surgery

sj-docx-2-cms-10.1177_12034754241266187 – Supplemental material for Management of Guttate Psoriasis: A Systematic Review Supplemental material, sj-docx-2-cms-10.1177_12034754241266187 for Management of Guttate Psoriasis: A Systematic Review by Ted Zhou, John Koussiouris, Lauren Kim and Ronald Vender in Journal of Cutaneous Medicine and Surgery

sj-docx-3-cms-10.1177_12034754241266187 – Supplemental material for Management of Guttate Psoriasis: A Systematic Review Supplemental material, sj-docx-3-cms-10.1177_12034754241266187 for Management of Guttate Psoriasis: A Systematic Review by Ted Zhou, John Koussiouris, Lauren Kim and Ronald Vender in Journal of Cutaneous Medicine and Surgery

sj-docx-4-cms-10.1177_12034754241266187 – Supplemental material for Management of Guttate Psoriasis: A Systematic Review Supplemental material, sj-docx-4-cms-10.1177_12034754241266187 for Management of Guttate Psoriasis: A Systematic Review by Ted Zhou, John Koussiouris, Lauren Kim and Ronald Vender in Journal of Cutaneous Medicine and Surgery

sj-docx-5-cms-10.1177_12034754241266187 – Supplemental material for Management of Guttate Psoriasis: A Systematic Review Supplemental material, sj-docx-5-cms-10.1177_12034754241266187 for Management of Guttate Psoriasis: A Systematic Review by Ted Zhou, John Koussiouris, Lauren Kim and Ronald Vender in Journal of Cutaneous Medicine and Surgery

sj-docx-6-cms-10.1177_12034754241266187 – Supplemental material for Management of Guttate Psoriasis: A Systematic Review Supplemental material, sj-docx-6-cms-10.1177_12034754241266187 for Management of Guttate Psoriasis: A Systematic Review by Ted Zhou, John Koussiouris, Lauren Kim and Ronald Vender in Journal of Cutaneous Medicine and Surgery

sj-docx-7-cms-10.1177_12034754241266187 – Supplemental material for Management of Guttate Psoriasis: A Systematic Review Supplemental material, sj-docx-7-cms-10.1177_12034754241266187 for Management of Guttate Psoriasis: A Systematic Review by Ted Zhou, John Koussiouris, Lauren Kim and Ronald Vender in Journal of Cutaneous Medicine and Surgery

sj-docx-8-cms-10.1177_12034754241266187 – Supplemental material for Management of Guttate Psoriasis: A Systematic Review Supplemental material, sj-docx-8-cms-10.1177_12034754241266187 for Management of Guttate Psoriasis: A Systematic Review by Ted Zhou, John Koussiouris, Lauren Kim and Ronald Vender in Journal of Cutaneous Medicine and Surgery
