# Supplementary material for: Management of Guttate Psoriasis: A Systematic Review
Source: J Cutan Med Surg. 2024 Jul 30;28(6):577–84. doi: 10.1177/12034754241266187 (PMC11619194; doi:10.1177/12034754241266187)
Supplement: sj-docx-1-cms-10.1177_12034754241266187 – Supplemental material for Management of Guttate Psoriasis: A Systematic Review [file sj-docx-1-cms-10.1177_12034754241266187.docx]

Figure S1. PRISMA Flow Diagram

**PRISMA Flow Diagram**

Records removed *before screening*:

Records marked as duplicates by automation tools and manually removed (n = 707)

Records identified from:

Medline (n = 429)

Embase (n = 1081)

CINAHL (n = 69)

Web of Science (n = 510)

**Total (n = 2089)**

**Identification**

Records title/abstract screened

(n = 1382)

Records excluded

(n = 1167)

**Screening**

Records excluded

(n = 140)

Reasons for exclusion:

- Did not report on guttate psoriasis treatment outcome data (n = 63)
- Did not investigate any treatments for guttate psoriasis

(n = 13)

- Only abstract/conference proceeding (n = 55)
- Not in English (n = 9)

Records full-text screened

(n = 215)

**Included**

Studies included in final review

(n = 75)
