# Supplementary material for: Management of Guttate Psoriasis: A Systematic Review
Source: J Cutan Med Surg. 2024 Jul 30;28(6):577–84. doi: 10.1177/12034754241266187 (PMC11619194; doi:10.1177/12034754241266187)
Supplement: sj-docx-2-cms-10.1177_12034754241266187 – Supplemental material for Management of Guttate Psoriasis: A Systematic Review [file sj-docx-2-cms-10.1177_12034754241266187.docx]

Supplemental Table S1. Full Search Strategy

*Medline*

| **Concept** | **Search Terms** |
| --- | --- |
| Psoriasis | 1. “psoriasis” OR “exp Psoriasis /” OR “exp Streptococcal Infections/” OR “exp Interleukin-17/” |
| Guttate Psoriasis | 1. “Guttate*” |
|  | 1. **1 AND 2 (n = 429)** |

*Embase*

| **Concept** | **Search Terms** |
| --- | --- |
| Psoriasis | 1. “psoriasis” OR “exp psoriasis/” OR “exp Streptococcal Infections/” OR “exp interleukin 17/” |
| Guttate Psoriasis | 1. “Guttate*” OR “exp guttate psoriasis/” |
|  | 1. **1 AND 2 (n = 1030)** |

*Web of Science*

| **Concept** | **Search Terms** |
| --- | --- |
| Psoriasis | 1. “psoriasis (All Fields)” OR “streptococcal infection* (All Fields)” OR “interleukin-17 (All Fields)” |
| Guttate Psoriasis | 1. “guttate*” |
|  | 1. **1 AND 2 (n = 510)** |

*CINAHL*

| **Concept** | **Search Terms** |
| --- | --- |
| Psoriasis | 1. “psoriasis” OR (MH “Psoriasis+”) OR (MH “Streptococcal Infections+”) OR (MH “Interleukins+”) |
| Guttate Psoriasis | 1. “guttate*” |
|  | 1. **1 AND 2 (n = 69)** |
