# Supplementary material for: Management of Guttate Psoriasis: A Systematic Review
Source: J Cutan Med Surg. 2024 Jul 30;28(6):577–84. doi: 10.1177/12034754241266187 (PMC11619194; doi:10.1177/12034754241266187)
Supplement: sj-docx-3-cms-10.1177_12034754241266187 – Supplemental material for Management of Guttate Psoriasis: A Systematic Review [file sj-docx-3-cms-10.1177_12034754241266187.docx]

**Supplemental Table S2.** Characteristics and Data Items Extracted from Included Studies

| **Topic** | **Data Items** |
| --- | --- |
| **Study Characteristics** | 1. Title 2. Authors 3. Year of Publication 4. Geographic Location 5. Type of Publication / Study |
| **Demographic Information of Participants** | 1. Age 2. Sex |
| **Treatment Response** | 1. Outcome Measurement Tool    1. i.e. Psoriasis Area Severity Index 2. Type of Intervention    1. Intervention and control groups if applicable 3. Magnitude of Improvement 4. Response Time 5. Follow-up    1. Follow-up time    2. Relapse? 6. Number of participants with progression to chronic plaque psoriasis 7. Summary Findings |
