# Supplementary material for: Management of Guttate Psoriasis: A Systematic Review
Source: J Cutan Med Surg. 2024 Jul 30;28(6):577–84. doi: 10.1177/12034754241266187 (PMC11619194; doi:10.1177/12034754241266187)
Supplement: sj-docx-4-cms-10.1177_12034754241266187 – Supplemental material for Management of Guttate Psoriasis: A Systematic Review [file sj-docx-4-cms-10.1177_12034754241266187.docx]

Supplemental Table S3. Treatment Outcomes Assessed Using Physician’s Judgement

| **Treatment** | **Total**  **Studies** | **Magnitude of Treatment Response***  **(# of Participants)** | | | | **Time of Final Response** | | **Follow-Up** | |
| --- | --- | --- | --- | --- | --- | --- | --- | --- | --- |
|  |  | SR | PR | NR | NS | Mean | Range | Patients | Relapse? |
| **Topical Corticosteroids** | 11 | 9 | 2 |  |  | 0.87 mths | 0.46 - 2 mths | 4 | 1 |
| *Adult*  *Pediatric* | 8  3 | 6  3 | 2 |  |  | 1.22 mths  0.67 mths | 0.46 - 2 mths  0.46 - 0.69 mths | 2  2 | 1  No |
| **Systemic Corticosteroid** | 2 | 1 | 1 |  |  | 0.75 mth | 0.5 – 0.8 mth | 0 |  |
| *Adult* | 2 | 1 | 1 |  |  | 0.75 mth | 0.5 - 0.8 mth | 0 | N/A |
| **Unspecified Antibiotic** | 1 | 1 |  |  |  | 0.5 mths |  | 0 |  |
| *Pediatric* | 1 | 1 |  |  |  | 0.5 mths | N/A | 0 | N/A |
| **Erythromycin** | 3 | 5 |  |  |  | 1 mth | 1 - 1.25 mths | 2 |  |
| *Pediatric* | 3 | 5 |  |  |  | 1 mth | 1 - 1.25 mths | 2 | No |
| **Penicillin** | 3 | 5 |  |  |  | 0.6 mths | 0.25 - 1 mth | 2 |  |
| *Pediatric* | 3 | 5 |  |  |  | 0.6 mths | 0.25 - 1 mth | 2 | No |
| **Penicillin + Erythromycin** | 2 | 2 |  |  |  | 1 mth |  | 0 |  |
| *Pediatric* | 2 | 2 |  |  |  | 1 mth | N/A | 0 | N/A |
| **Doxycycline** | 1 | 1 |  |  |  | NS |  | 1 |  |
| *Pediatric* | 1 | 1 |  |  |  | NS | N/A | 1 | No |
| **Topical Corticosteroids + Methotrexate** | 2 | 2 |  |  |  | 2.69 mths | 1.38 - 4 mths | 2 |  |
| *Adult* | 2 | 2 |  |  |  | 2.69 mths | 1.38 - 4 mths | 2 | No |
| **Methotrexate** | 3 | 1 | 1 |  | 1 | 1 mth | 0.5 - 1.5 mths | 1 |  |
| *Adult* | 3 | 1 | 1 |  | 1 | 1 mth | 0.5 - 1.5 mths | 1 | No |
| **Cyclosporine + Oral Antibiotic** | 1 | 1 |  |  |  | 0.25 mth |  | 0 |  |
| *Adult* | 1 | 1 |  |  |  | 0.25 mth | N/A | 0 | N/A |
| **Tonsillectomy** | 2 | 8 | 1 |  |  |  |  | 1 |  |
| *Adult*  *Pediatric*  *Unspecified* | 1  1  1 | 1  2  5 | 1 |  |  | NS  2 mths  NS | N/A  N/A  N/A | 1  N/A  N/A | No  N/A  N/A |
| **Corticosteroid + Coal Tar** | 1 | 1 |  |  |  | 0.69 mths |  | 1 |  |
| *Pediatric* | 1 | 1 |  |  |  | 0.69 mths | N/A | 1 | No |
| **IVIG** | 1 | 1 |  |  |  | 4 mths |  | 1 | 1 |
| *Pediatric* | 1 | 1 |  |  |  | 4 mths | N/A | 1 | 1 |
| **Phototherapy + Penicillin** | 1 | 1 |  |  |  | 1.84 mths |  | 1 |  |
| *Pediatric* | 1 | 1 |  |  |  | 1.84 mths | N/A | 1 | No |
| **Salicylic Acid** | 1 | 1 |  |  |  | 0.69 mths |  | 0 |  |
| *Pediatric* | 1 | 1 |  |  |  | 0.69 mths | N/A | 0 | N/A |
| **Calcipotriol + Corticosteroid** | 4 | 3 | 1 |  |  | 0.69 mths |  | 0 |  |
| *Adult*  *Pediatric* | 2  2 | 1  2 | 1 |  |  | 0.69 mths  2 mths | N/A  N/A | 0  0 | N/A  N/A |
| **Biologics** | 4 | 8 |  |  | 1 | 3.27 mths | 0.9 - 6.9 mths | 8 | 2 |
| *Adult* | 4 | 8 |  |  | 1 | 3.27 mths | 0.9 - 6.9 mths | 8 | 2 |
| **Autologous SCT** | 1 | 1 |  |  |  | 0.66 mths |  | 1 |  |
| *Pediatric* | 1 | 1 |  |  |  | 0.66 mths | N/A | 1 | No |
| **Calcipotriene Cream** | 1 | 1 |  |  |  | 0.46 mths |  | 0 |  |
| *Adult* | 1 | 1 |  |  |  | 0.46 mths | N/A | 0 | N/A |
| **Phototherapy + Corticosteroid** | 4 | 4 |  |  |  | 0.69 mths |  | 3 | 1 |
| *Adult*  *Pediatric* | 3  1 | 3  1 |  |  |  | 0.69 mths  0.69 mths | N/A  N/A | 3  0 | 1  N/A |
| **Antifungal** | 1 | 1 |  |  |  | 0.46 mths |  | 0 |  |
| *Adult* | 1 | 1 |  |  |  | 0.46 mths | N/A | 0 | N/A |
| **Phototherapy + Retinoids** | 1 | 1 |  |  |  | 2 mths |  | 0 |  |
| *Adult* | 1 | 1 |  |  |  | 2 mths | N/A | 0 | N/A |

** SR = Significant or Marked Response (Defined by physician-described improvement), PR = Partial or Moderate Response (Defined by physician-described improvement), NR = Minimal or No Response (Defined by physician-described improvement), NS = Not Specified, M = Male, F = Female*
