# Supplementary material for: Management of Guttate Psoriasis: A Systematic Review
Source: J Cutan Med Surg. 2024 Jul 30;28(6):577–84. doi: 10.1177/12034754241266187 (PMC11619194; doi:10.1177/12034754241266187)
Supplement: sj-docx-5-cms-10.1177_12034754241266187 – Supplemental material for Management of Guttate Psoriasis: A Systematic Review [file sj-docx-5-cms-10.1177_12034754241266187.docx]

Supplemental Table S4. Treatment Outcomes Assessed Using Outcome Measurement Tools (PASI, BSA, PGA)

| **Treatment** | **Total**  **Studies** | **Magnitude of Treatment Response***  **(# of Participants)** | | | | **Time of Final Response** | | **Follow-Up** | |
| --- | --- | --- | --- | --- | --- | --- | --- | --- | --- |
|  |  | SR | PR | NR | NS | Mean | Range | Patients | Relapse? |
| **Guselkumab** | 2 | 1 |  | 1 |  | 4.5 mths | 1 - 8 mths | 1 |  |
| *Adult* | 2 | 1 |  | 1 |  | 4.5 mths | 1 - 8 mths | 1 | No |
| **Risankizumab** | 2 | 5 |  |  |  | 2.2 mths | 1 - 4 mths | 4 |  |
| *Adult* | 2 | 5 |  |  |  | 2.2 mths | 1 - 4 mths | 4 | No |
| **Secukinumab** | 2 | 3 |  |  |  | 4 mths | N/A | 1 |  |
| *Adult* | 2 | 3 |  |  |  | 4 mths | N/A | 1 | No |
| **Ixekizumab** | 2 | 4 |  |  |  | 1.81 mths | 0.5 - 3 mths | 4 |  |
| *Adult* | 2 | 4 |  |  |  | 1.81 mths | 0.5 - 3 mths | 4 | No |
| **Phototherapy + Methoxsalen** | 1 | 1 |  |  |  | NS |  | 1 |  |
| *Pediatric* | 1 | 1 |  |  |  | NS | N/A | 1 | No |

** SR = Significant or Marked Response (Defined as* ≥ *75% improvement), PR = Partial or Moderate Response (Defined as* ≥*25% - <75% improvement), NR = Minimal or No Response (Defined as <25% improvement), NS = Not Specified, M = Male, F = Female, PASI = Psoriasis Area and Severity Index, BSA = Body Surface Area, PGA = Physician Global Assessment*
