# Supplementary material for: Management of Guttate Psoriasis: A Systematic Review
Source: J Cutan Med Surg. 2024 Jul 30;28(6):577–84. doi: 10.1177/12034754241266187 (PMC11619194; doi:10.1177/12034754241266187)
Supplement: sj-docx-6-cms-10.1177_12034754241266187 – Supplemental material for Management of Guttate Psoriasis: A Systematic Review [file sj-docx-6-cms-10.1177_12034754241266187.docx]

Supplemental Table S5. Summary of Randomized Controlled Trials

| **Study** | **Population** | **Intervention** | **Control** | **Outcome** | **Response Time** | **Results** |
| --- | --- | --- | --- | --- | --- | --- |
| Boztepe et al., 2006 | 14 pediatric and adult GP patients after achieving clinically significant improvement | 2 months of narrow-band (311 nm) UVB maintenance | No maintenance | PASI | 1 year | The number of patients in remission at the end of the study were four of eight (50%) and five of six (83%) in the maintenance and no maintenance groups, respectively. There was no statistically significant difference between the two groups. |
| Caca-Biljanovska et al., 2002 | 20 adult GP patients | Betamethasone dipropionate 0.05% cream plus UVB  with penicillin | Betamethasone dipropionate 0.05% cream plus UVB  without penicillin | PASI | 8 weeks | There was no significant difference between the PASI values for the two subgroups at the end of treatments. No significant difference in the effectiveness of two therapies. |
| Dogan et al., 2008 | 43 adult GP patients | Group 1 = oral erythromycin 250 mg four times a day for 14 d.  Group 2 = oral benzathine phenoxymethylpenicillin 50 000 IU/kg/d  divided in three doses for 14 d | No treatment group | PASI | 4 weeks | No statistically significant clinical improvement was detected in either treatment or control  groups at the end of the treatment and at  4-week follow-up. |
| Grimminger et al., 1993 | 20 adult GP patients | n-3 fatty acid based lipid emulsion [100 ml/day with 2.1 g eicosapentaenoic (EPA) and 21 g docosahexaenoic acid (DHA)] | Conventional n-6 lipid emulsion (EPA + DHA < 0.1 g/100 ml) | Physician's Judgment | 10 days | Moderate improvement was noted in the n-6  group (16-25% from baseline within 10 days). Significant improvement was noted in the n-3 group (45% and 76% within 10 days). |
| Tsankov et al., 2011 | 92 adult GP patients | Oral rifampicin  (600 mg daily dose for 60 days) and topical emollients | Placebo capsules in same daily dosage for 60 days, and emollients | PASI | 60 days | The efficacy of rifampicin compared with placebo was significantly higher. No significant difference in the therapeutic response to rifampicin in patients with guttate psoriasis with or without concomitant infection. |
