# Supplementary material for: Management of Guttate Psoriasis: A Systematic Review
Source: J Cutan Med Surg. 2024 Jul 30;28(6):577–84. doi: 10.1177/12034754241266187 (PMC11619194; doi:10.1177/12034754241266187)
Supplement: sj-docx-7-cms-10.1177_12034754241266187 – Supplemental material for Management of Guttate Psoriasis: A Systematic Review [file sj-docx-7-cms-10.1177_12034754241266187.docx]

Supplemental Table S6. Risk of Bias Assessment for Included Non-Randomized Studies (ROBINS-I)

| **Study** | **D1** | **D2** | **D3** | **D4** | **D5** | **D6** | **D7** | **Overall** |
| --- | --- | --- | --- | --- | --- | --- | --- | --- |
| Gunther, 1973 | Moderate | Low | Low | Moderate | Low | Moderate | Low | Moderate |
| Petrozzi et al., 1997 | Moderate | Moderate | Low | Low | Low | Moderate | Moderate | Moderate |
| Gomez et al., 1996 | Moderate | Moderate | Low | Moderate | Moderate | Moderate | Low | Moderate |
| Masood et al., 2000 | Low | Moderate | Low | Serious | Serious | Moderate | Moderate | Serious |
| Gokdemir et al., 2005 | Moderate | Moderate | Low | Moderate | Moderate | Moderate | Moderate | Moderate |
| Hollo et al., 2005 | Moderate | Moderate | Low | Moderate | Moderate | Moderate | Moderate | Moderate |
| Carvalho et al., 2013 | Critical | Low | Moderate | Critical | Low | Serious | Low | Critical |
| Fernandez-Guarino et al., 2016 | Moderate | Moderate | Low | Moderate | Moderate | Moderate | Moderate | Moderate |
| Farrell et al., 2021 | Serious | Moderate | Low | Serious | Moderate | Serious | Low | Serious |

*D1: Bias due to confounding, D2: Bias in selection of participants into the study, D3: Bias in classification of interventions, D4: Bias due to deviations from intended interventions, D5: Bias due to missing data, D6: Bias in measurement of outcomes, D7: Bias in selection of the reported result*
