# Supplementary material for: Management of Guttate Psoriasis: A Systematic Review
Source: J Cutan Med Surg. 2024 Jul 30;28(6):577–84. doi: 10.1177/12034754241266187 (PMC11619194; doi:10.1177/12034754241266187)
Supplement: sj-docx-8-cms-10.1177_12034754241266187 – Supplemental material for Management of Guttate Psoriasis: A Systematic Review [file sj-docx-8-cms-10.1177_12034754241266187.docx]

Supplemental Table S7. Risk of Bias Assessment for Included Randomized Controlled Trials (RoB 2.0)

| **Study** | **D1** | **D2** | **D3** | **D4** | **D5** | **Overall** |
| --- | --- | --- | --- | --- | --- | --- |
| Grimminger et al., 1993 | Some | Some | Low | High | Some | High |
| Caca-Biljanovska et al., 2002 | Low | Low | Low | Some | Some | Some |
| Boztepe et al., 2006 | High | High | High | Some | Some | High |
| Dogan et al., 2008 | Some | High | High | Low | Some | High |
| Tsankov et al., 2011 | High | Low | Low | High | Some | High |

*D1: Bias arising from randomization process, D2: Bias due to deviations from the intended interventions, D3: Bias due to missing outcome data, D4: Bias in measurement of the outcome, D5: Bias in selection of the reported result*
